# Friend or foe: How plants discriminate between pathogenic and mutualistic bacteria

**DOI:** 10.1093/plphys/kiac238

**Published:** 2022-05-24

**Authors:** Bernarda Calla

**Affiliations:** Forage Seed and Cereal Research Unit, USDA-ARS, Corvallis, Oregon, 97331, USA

Legumes can form symbiotic associations with rhizobia, which are nitrogen-fixing bacteria that colonize roots by forming facultative organs termed nodules. The development of nodules depends on a concerted interchange of signals between the plant and the symbiotic bacteria. Ultimately, legumes benefit from this association by being able to use nitrogen that would otherwise be unavailable, while rhizobia successfully multiply by utilizing plant resources mainly in the form of sugars (Oldroyd et al., 2011). The question remains, however, how plants distinguish these beneficial bacteria from bacteria that cause disease.

Over a decade ago, research showed that some symbiotic bacteria could elicit the plant immune system by introducing specific proteins into host plants in a manner reminiscent of pathogenic bacteria (Deakin and Broughton, 2009). These proteins, known as nodulation outer proteins (Nops), can either induce nodulation or inhibit nodulation in a host-specific fashion, just as in the case of pathogen virulence and avirulence factors. Few plant proteins have been shown to interact directly or indirectly with Nops to trigger those responses and the mechanism of Nops action is poorly understood (Staehelin and Krishnan, 2015).

In this issue of *Plant Physiology*, Khan and collaborators (2022) investigate the mode of action of NopT from *Sinorhizobium* (*Ensifer*) strain NGR234. This rhizobium strain can nodulate species from more than 100 plant genera (Pueppke and Broughton, 1999). NopT induces nodulation in strains of common bean (*Phaseolus vulgaris*) and negatively affects nodulation in the tropical legume *Crotalaria pallida* (Dai et al., 2008). Once in the plant, NopT cleaves itself to expose residues that become lipidated, activating a localization signal that directs NopT to the plasma membrane (Dowen et al., 2009).

The authors first utilized a Xanthomonas (*Xanthomonas campestris*)–pepper (*Capsicum annuum*) translocation system to show that translocation of the NopT effector from NGR234 occurs through a bacterial type III secretion system (T3SS) and depends on NopT N-terminal residues. Further, with the use of a NGRΔ*nopT* mutant and by introducing plasmids with *nopT* mutations that lack specific residues, the authors corroborated that NopT requires lipidation and its protease activity to inhibit nodulation in *C. pallida*. To test the contribution of NopT in nodulation, the authors introduced *nopT* into *Sinorhizobium fredii* strain USDA257, which can induce nodulation in a wide range of soybean (*Glycine max*) accessions. The authors showed that *nopT* of NGR234 in the USDA257 strain inhibited nodulation of soybean cv. Nenfeng 15, indicating that NopT alone is sufficient to prevent symbiosis in some soybean accessions and when expressed in USDA257.

Next, the authors investigated how NopT interferes with nodulation and their findings indicate that NopT triggers the plant defense pathway known as effector-triggered immunity (ETI). Plants have evolved an innate immune system that swiftly recognizes the majority of pathogens in their environment to mount a defensive response. In this innate immune system, a plant recognizes pathogen-associated molecular patterns to elicit a general response known as “pathogen triggered immunity” (PTI). In turn, some pathogens suppress PTI by secreting effectors, to which the plant may or may not be able to respond by triggering ETI, a second layer of response. ETI is strain-specific and characterized by hypersensitive response and programmed cell death at the site of infection, which effectively restrict pathogen proliferation (Jones and Dangl, 2006).

NopT has sequence and structural homology with the avirulence factor AvrPphB from the pathogenic *Pseudomonas syringae* (pv*. phaseolicola*). AvrPphB is an effector protease that can trigger ETI by cleaving the plant cytoplasmatic receptor-like kinase “AvrPphB Susceptible1” (PBS-1), which in turn activates the resistance protein “Resistance to Pseudomonas Syringae 5” (*RPS5*) (Ade et al., 2007). Khan and collaborators show that NopT functions like AvrPphB. *Agrobacterium tumefaciens*-mediated transformation of *nopT* of NGR234 into wild-type Arabidopsis (*Arabidopsis thaliana*) resulted in chlorosis reminiscent of the hypersensitive response, whereas transient expression of NopT in *A. thaliana* mutants *pbs1-2* and *rps5-2* did not show any response (Figure 1A). Further, the authors co-expressed tagged NopT-HA with AtPBS1-FLAG or soybean GmPBS1-1-FLAG and showed that NopT effectively cleaves both PBS1 proteins (Figure 1B).

**Figure 1:**
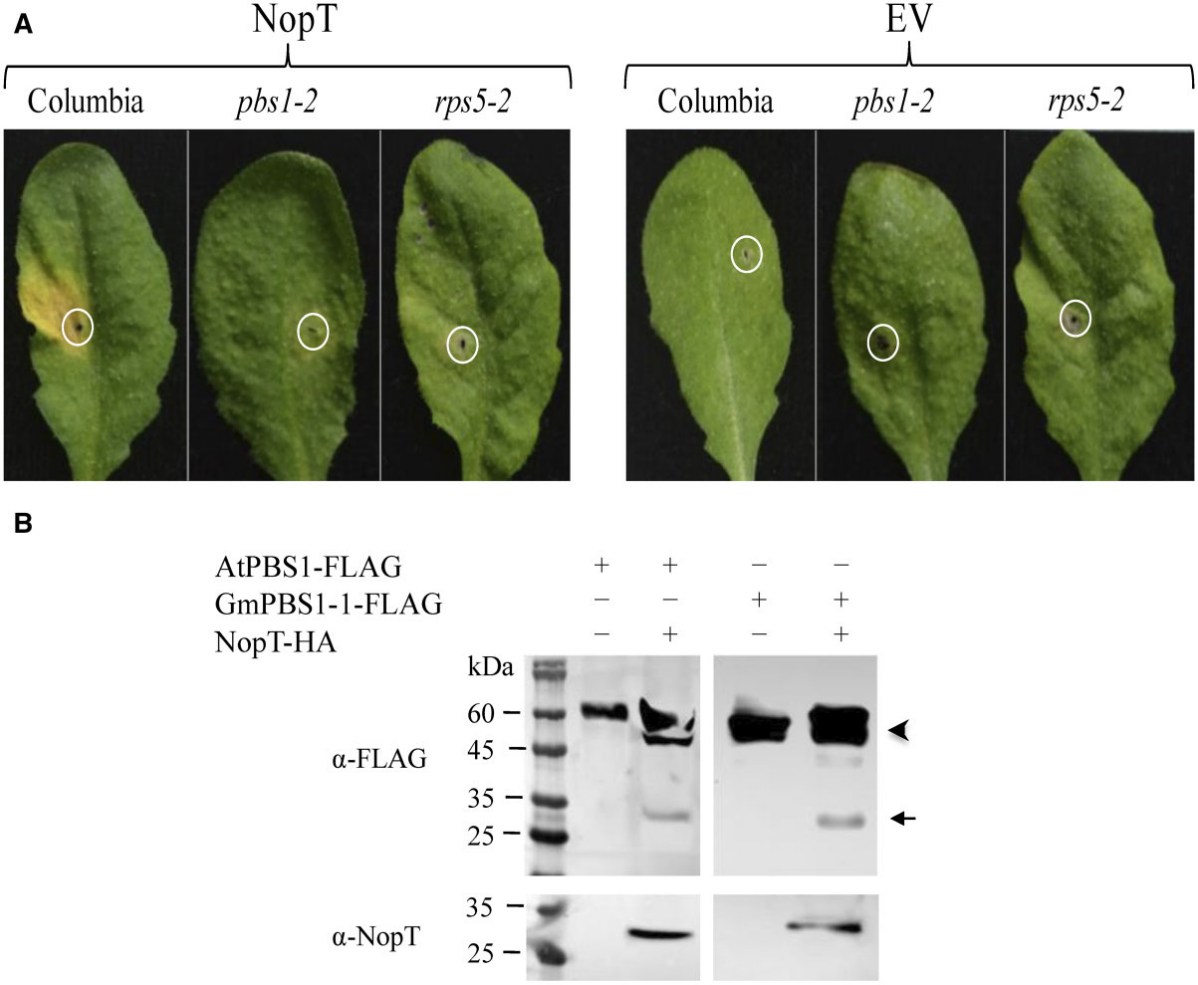
NopT of *Sinorhizobium* (*Ensifer*) strain NGR234 functions like AvrRPphB from *P. syringae* in triggering an ETI-like hypersensitive response that involves PBS1 and RPS5. A, Agroinfiltration in *A. thaliana* leaves shows that NopT triggers chlorosis in wild-type but not in *pbs1-2* and *rps5-2* leaves. B, Protein expression of NopT-HA with AtPBS1-FLAG or GmPBS1-1-FLAG demonstrates that NopT cleaves PBS1 proteins. AtPBS1-FLAG and GmPBS1-1-FLAG are marked by an arrowhead and the FLAG-tagged cleavage products by an arrow. Modified from Khan et al. (2022). EV, empty vector; At, *Arabidopsis thaliana*; and Gm, soybean.

This study shows how an effector from symbiotic bacteria is recognized in the plant as a pathogen effector to trigger protein cleavage, leading to a plant defense response. Moreover, this recognition is strain-specific since soybean plants from different strains can be either compatible or incompatible with NGR234. Since PBS1 proteins are conserved in soybean, compatibility or incompatibility might be determined by one or more additional proteins that, like RPS5 in the plant response to pathogenic *P. syringae* AvrPphB, react to the cleavage of PBS1. A next step in this quest would be the identification of such protein(s).

The plant immune system seems to walk a fine line in discriminating pathogens from symbionts. Evolutionarily, host plants are faced with the challenge between allowing infection to proceed or defending against it; whereas bacteria, either pathogenic or symbiotic, share the similar evolutionary goal of colonizing the plant cell to their advantage. This study provides insight into an additional step in effector-triggered plant responses where the line between pathogenicity and symbiosis might be drawn.

## Disclaimer

The author contributed to this article in her personal capacity. The views and opinions expressed within are those of the author and do not necessarily represent the views of the Agricultural Research Service, USDA, or the United States Government.

*Conflict of interest statement*. None declared.
